# Bimanual attempts improving EEG-based hand movement decoding in stroke patients with hemiplegia

**DOI:** 10.3389/fnhum.2026.1890774

**Published:** 2026-07-28

**Authors:** Jiarong Wang, Luzheng Bi, Weijie Fei, Yuyang Wei

**Affiliations:** The School of Mechanical Engineering, Beijing Institute of Technology, Beijing, China

**Keywords:** bimanual movement, brain–computer interface, motor attempt, neural decoding, neural signatures, stroke patients

## Abstract

**Background:**

Brain-computer interfaces (BCIs) based on electroencephalography (EEG) signals have been applied to improve active hand motor function rehabilitation of stroke patients. Besides, the bilateral arm rehabilitation training can effectively promote the recovery of the unilateral affected hand. However, existing studies on BCIs for stroke patients mainly focus on decoding the unilateral affected hand without effectively utilizing the unaffected hand.

**Objective:**

In this paper, we studied the neural signatures and decoding of bimanual and unimanual motor attempts of hemiplegic stroke patients from EEG signals to explore the neural signature differences between bimanual and unimanual movements of patients and whether the differences can improve movement decoding accuracy. Methods: We designed the experimental paradigm of unimanual and bimanual opening and closing motor attempts. Furthermore, we compared the neural signature differences using movement-related cortical potential (MRCP) and event-related desynchronization (ERD).

**Results:**

Experimental results from hemiplegic stroke patients indicated that greater MRCP activation was found during bimanual movements than unimanual movements. Although the event-related spectral perturbation (ERSP) varied among patients, it mainly appeared in the [8–30] Hz frequency band, and the ERD activation of bimanual movements was greater than that of unimanual movements in specific frequency bands and brain regions for each patient. The average decoding accuracy of bimanual movements was higher than that of unimanual movements by 4% to 12%.

**Discussion:**

This work can potentially advance BCI-based active rehabilitation of hand motor function of stroke patients.

## 1 Introduction

Stroke is a severe disease of neurological damage, and it has become a leading cause of adult disabilities in the world, with approximately 30% of survivors suffering from motor disability due to stroke (Benjamin et al., 2019). The deficit in hand function seriously impairs the quality of patients lives. In severe cases, the patients may be unable to perform activities of daily living (ADL), such as dressing, feeding, and self-care (Schwarz et al., 2020). The main techniques for recovering stroke-related motor deficits include physical and robotic therapies. However, these two therapies are passive for patients without residual movement (Ramos-Murguialday et al., 2013).

Brain–compute interface (BCI) is a new communication way established between the brain and external devices, bypassing peripheral nerves and muscles (Millan et al., 2010). For patients without residual movement or with slight residual movement, BCI has been proven to promote motor recovery effectively (Yang et al., 2022; Sitaram et al., 2012). Existing studies decoded patients movement intentions based on motor imagery (MI) and motor attempt (MA) paradigm. In 2016, Antelis et al. (2017) achieved significant continuous decoding of paralyzed upper-limb MA from electroencephalography (EEG) signals on the contralesional motor cortex. In 2019, Ofner et al. (2019) extracted amplitudes of low-frequency EEG signals to decode five classes of MA tasks (hand open, palmar grasp, lateral grasp, pronation, and supination) and obtained a maximum average classification accuracy of 45%. In 2020, Benzy et al. (2020) decoded the direction (left vs. right) of patients affected arms based on MI tasks and achieved an average binary accuracy of 68.63% across subjects. In 2020, Lu et al. (2020) studied the efficiency of using BCI based on MI task to control continuous passive movements in wrist extension recovery. Through continuous BCI-driven training, the active motion range of the affected wrist has been increased. In 2021, Lee et al. Lee et al. (2021) used EEG signals to decode a finger-tapping task with an affected hand in stroke patients during an MA task (except for the thumb, moving the other four fingers separately) and obtained an average four-class accuracy of 46.94%.

Meanwhile, numerous studies have shown that bilateral movement training improves motor recovery. In 2000, Byblow et al. (2000) found that the performance of the impaired limb was enhanced when the impaired limb was coupled in phase with the unimpaired limb. In 2003, Cauraugh and Kim (2003) showed a significant promoting effect on wrist extension movement of the affected limb when bimanual moved simultaneously. In 2020, Renner et al. (2020) compared the unilateral and bilateral arm training effects on arm impairment in severely affected stroke patients, and results showed a significantly greater improvement following the bilateral training in Fugl–Meyer Assessment arm score compared with unilateral training. From the neurophysiological perspective, bilateral movements activated similar neural networks in bilateral hemispheres, and the unaffected hemisphere was activated during bilateral movement, which increased the activation of the damaged hemisphere (Wenderoth et al., 2004; Hallett, 2001). It can explain why bilateral movements promote motor recovery.

Existing studies showed bilateral training was effective for motor recovery and active motor rehabilitation of impaired hand based on BCI techniques was feasible (Liao et al., 2023). Besides, bimanual cooperation movements are essential in ADL, and the motor coordination functions could also be improved by bilateral training. However, to our knowledge, there is no research on neural signatures and movement decoding of bimanual movements from EEG signals for hemiplegic patients to date, and whether bimanual motor attempts can improve hand movement decoding performance for hemiplegic patients remains open.

Thus, in this work, our main objective is to investigate the neural signatures and decoding of bimanual motor attempts of hemiplegic stroke patients based on EEG signals. We aim to explore neural signature differences between bimanual and unimanual movements of patients and whether the differences can improve decoding accuracy. The contribution of this paper is that it is the first to explore the neural signature differences between motor attempts of bimanual and unimanual of hemiplegic stroke patients based on EEG signals and whether the neural signature differences can enhance decoding performance. The findings can promote the progress of the BCI-based active rehabilitation of hand function of stroke patients.

## 2 Method

### 2.1 Experimental paradigm

The Medical Ethics Committee of Qingdao Traditional Chinese Medicine Hospital (Haiser Hospital) approved the experiment with the ethical number 2024HC01LS16. Ten stroke patients participated in the experiment. Two of ten patients failed to complete the experiment. Thus, eight patients (six male and two female) were involved for data analysis. The patients' recruitment criteria included: (i) unilateral brain lesion caused by stroke; (ii) affected hand unable to actively extend; (iii) no other neurological illnesses and cognitive impairments. The detailed information of patients is provided in Table 1. The study adhered to the principles of the 2013 Declaration of Helsinki.

**Table 1:** Demographic and clinical information of the patients.

Patients	Age (years)	Gender	Days since stroke	Hemisphere of lesion
P1	67	M	41	Right
P2	49	M	5	Left
P3	49	M	91	Right
P4	71	M	110	Left
P5	59	F	2	Left
P6	70	F	69	Right
P7	74	M	25	Right
P8	74	M	35	Right

**Figure 1:**
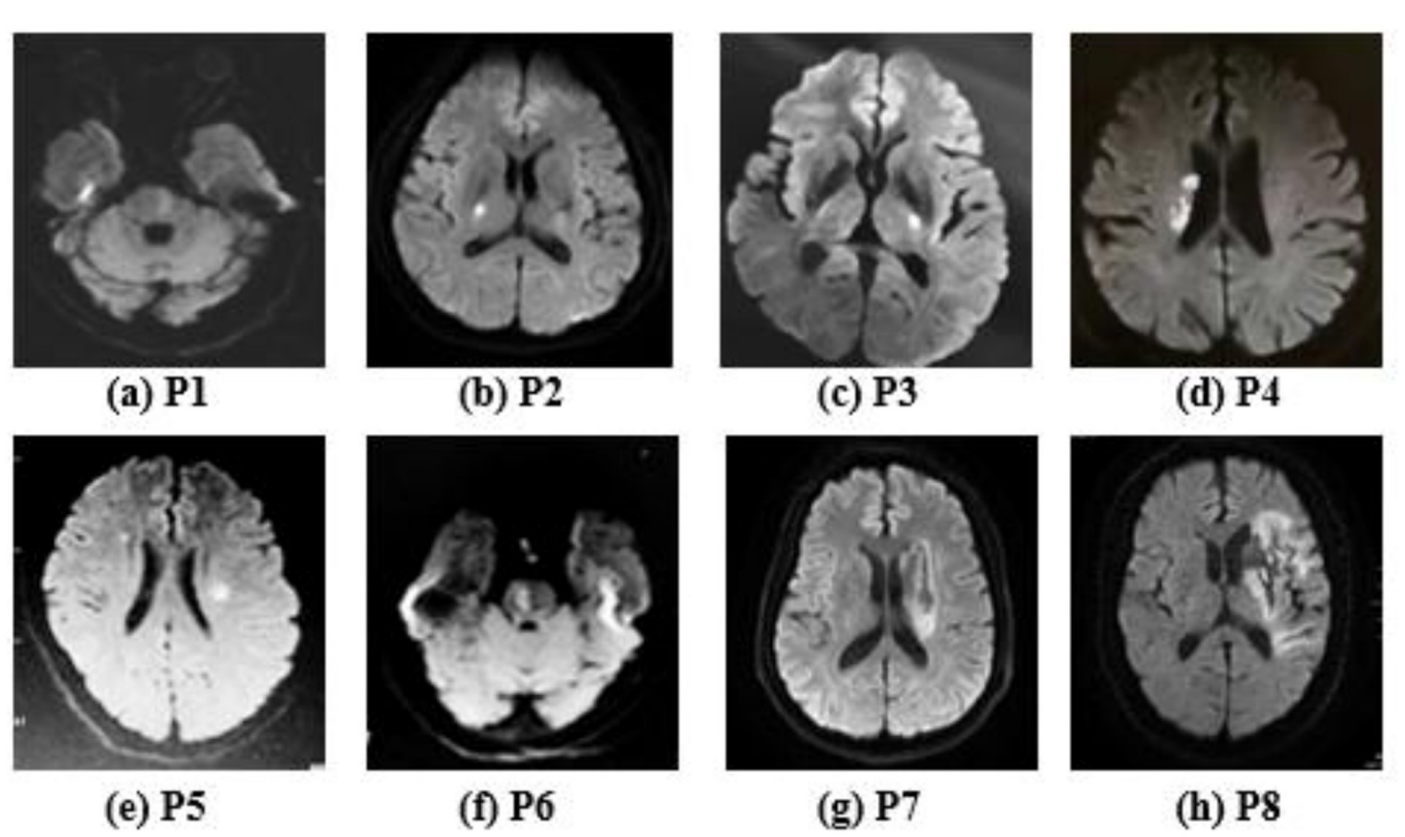
Cranial MRI images of eight stroke patients with hemiplegia. **(a)** P1. **(b)** P2. **(c)** P3. **(d)** P4. **(e)** P5. **(f)** P6. **(g)** P7. **(h)** P8.

The cranial magnetic resonance imaging (MRI) images of eight patients are shown in Figure 1, where the brightness enhancement area is the affected lesion area. The lesion areas were mainly concentrated in the basal ganglia and paraventricular regions, corresponding to the central area, which mainly affected the motor function of the upper limb.

In our experiment, the patients were required to perform unimanual and bimanual opening and closing movements. Patients placed their arms on a table and followed the screen stimuli to perform left-hand, right-hand, and bimanual movements. For the hemiplegic hand, we required the patients to perform MA. The experiment associated with each hand movement was composed of eight runs, with a rest period of 2 min in between. Each run consisted of ten trials, with five opening and five closing movements. Task order was counterbalanced for all participants to avoid sequence effects. Participants could take breaks on demand to relieve fatigue. Analysis of run-wise performance revealed no obvious drift, suggesting fatigue exerted little impact on the experimental outcomes.

At the beginning, there was no stimulus on the screen. The patients were asked to focus and relax their hands in a half-grip clench posture. At the fourth second, a picture of opening or closing the hand randomly appeared in the center of the screen, indicating patients to perform the hand movement. At the eighth second, patients were required to relax their hands and return to a half-grip clench when the ‘relax cue appeared on the screen. For the unimanual tasks, patients only performed motor attempts with the targeted hand, while the contralateral hand remained relaxed. For the bimanual tasks, both the left and right hands attempted to move. Each trial lasted for 13 s. Each type of movement contained 80 trials. The entire experiment lasted for approximately 1 h and 40 min.

### 2.2 Data acquisition and preprocessing

EEG signals were recorded by a 64-electrode portable wireless EEG amplifier (NeuSen.W64, Neuracle Technology Co., Ltd.) from the brain scalp of patients at the Fz, F1, F2, F3, F4, F5, F6, FCz, FC1, FC2, FC3, FC4, FC5, FC6, Cz, C1, C2, C3, C4, C5, C6, CP1, CP2, CP3, CP4, CP5, CP6, Pz, P3, P4, P5, P6, P7, P8, Oz, O1, O2 locations according to an international 10-20 system. For patients with left hemiplegia, we symmetrically exchanged EEG signals from both hemispheres so that all patients had the same hemiplegic limb (right hand) for the convenience of analysis. We defined the right hand and left hand as the ipsilateral affected hand and the contralateral unaffected hand, respectively.

The sampling rate of EEG signals was 1000 Hz. Electrode impedances were calibrated to be less than 5 kΩ. Reference electrode used linked mastoid mean reference. Two extra electrodes were used to collect electrooculogram (EOG) signals. We first down-sampled the signals to 100 Hz and used the baseline correction to remove the baseline drifts (Bi et al., 2021). The Nyquist frequency of 50 Hz exceeds the upper limit of our analyzed frequency band (40 Hz), preventing aliasing. This choice reduces computational cost for preprocessing and decoding while maintaining adequate temporal resolution for MRCP and 8–30 Hz ERD extraction, consistent with standard practices in stroke motor attempt BCI research. Raw EEG was first filtered with a 50 Hz notch filter for power-line interference removal and then filtered with a 0.1–40 Hz band-pass filtering. Artifact subspace reconstruction (ASR) was subsequently used to eliminate large-amplitude motion artifacts caused by body and head movement, which differ from EMG artifacts generated by facial muscle activity, where the cut-off threshold parameter *k* was set to 10. Then, the independent component analysis (ICA) was performed to separate signal sources. Independent components correlated with EOG signals above a 0.4 threshold were removed as ocular artifacts, and components characterized by broadband high-frequency power were recognized as EMG artifacts and excluded simultaneously (Wang et al., 2021). We further verified the efficacy of artifact removal by analyzing rejected ICA components. Components correlated with EOG and those showing typical EMG features were systematically excluded. Post-processing inspection on peripheral channels confirmed that ocular and muscular artifacts were largely eliminated. Participants were required to refrain from unnecessary body movements to reduce movement-induced confounding. All preprocessing, neural feature analysis and decoding experiments were completed uniformly in MATLAB.

### 2.3 Data analysis

The fourth second of each trial was defined as the movement cue onset. For the moving and rest periods, we selected the epoch [–2, 3] s of the movement cue onset for analysis. Then, we performed neural signatures analysis and movement intention decoding in time and frequency domains.

#### 2.3.1 Movement-related cortical potential (MRCP)

MRCP encodes movement-related information during motor planning and execution (Jochumsen et al., 2015). Therefore, we can use MRCPs to represent the neural activity during hand movements in time domain. We applied a fourth-order [0.01–4] Hz band-pass ButterWorth filter to extract low-frequency EEG signals.

#### 2.3.2 Event-related desynchronization (ERD)

Electrophysiological correlates of activated cortical areas which involved in processing of motor-related behavior can be represented by the reduction of alpha band power, known as ERD (Edelman et al., 2016). ERD can be demonstrated using event-related spectral perturbation (ERSP), which refers to the event-related changes in the EEG spectrum and is typically displayed in the two-dimensional time-frequency domain, and can be calculated as follows (Makeig et al., 2004):


$$\begin{array}{l} ERD=\frac{A-R}{R}\times 100\% \end{array}$$ (1)

where *A* is the power within the frequency band of interest in the period after the event and *R* is the preceding baseline or reference period.

We used the epoch [–3, –1] s as the baseline. According to Pfurtscheller and da Silva (1999), ERD was subject-specific. Thus, we calculated the ERSPs of each patient and used different frequency bands for different patients to calculate the ERD results. Different from the frequency band 0.01–4 Hz of MRCP, the preprocessed EEG spanning 0.1–40 Hz was used for ERD. The narrow low-frequency band isolates motion-related slow cortical potentials without high-frequency oscillation interference, whereas the full wideband decomposition preserves complete alpha and beta rhythm information needed to calculate ERD responses across subjects.

#### 2.3.3 Feature extraction and classification

For the preprocessed EEG signals, we extracted features for classification. We selected the epoch [–2, –1] s of movement cue onset as the samples with no movement intention. According to Wang et al. (2021), the amplitude features of MRCPs were extracted as temporal features. Each channel had 100 features. All amplitudes of the 37 channels were concatenated into a 3,700-dimension feature vector. To reduce the feature dimension, principal component analysis (PCA) was applied to reduce temporal features dimension to 40. Thus, the temporal feature was a 40-dimensional vector.

Common spatial pattern (CSP) was applied as spatial filters to extract features for classification in specific frequency bands. However, the classification performance mainly depends on the selected frequency band. To solve the issue of manually selecting specific frequency bands in the CSP algorithm, Ang et al. (2008) proposed a novel machine learning approach called filter bank common spatial pattern (FBCSP). The FBCSP algorithm consists of four stages: frequency filtering, spatial filtering, feature selection, and classification. In this work, we decomposed the EEG signals into ten frequency bands using a fourth-order band-pass Butterworth filter: 1–4, 4–8, 8–12, …, 32–36, 36-40 Hz. The pairs of CSP features were selected as 2. We applied the Mutual Information Best Individual Features (MIBIF) algorithm to select features. According to Ang et al. (2008), the value *k* was set to be 4, and the first four best features were selected as spectral features. Thus, the spectral features were a 4-dimensional vector. Linear discriminant analysis (LDA) classifier was used for movement classification. Samples with movement intention were selected with a window length of 1 s and a step size of 0.1 s after movement cue onset. Each window was analyzed independently with a 5 × 5 cross-validation. For each window, all corresponding samples were randomly split into five folds; four folds were for training and one for testing, and the whole procedure was repeated five times with re-splitting. Since cross-validation was executed separately for every window, there was no dependence between training and testing samples.

## 3 Results

Due to differences in cognitive and physical functions among patients, there were significant differences in response time to the movement cue (Pichierri et al., 2011). Furthermore, the location and condition of lesions varied among patients, leading to a certain degree of differences in neural signatures (Rocha et al., 2024). Thus, the grand average neural signature of all patients may not clearly encode subject-specific movement information. We chose a typical patient (Patient 8) to display neural signatures. The neural signatures of other patients were included in Supplementary material. All pairwise comparisons of MRCP and ERD minimum peak negativity across movement conditions were performed using two-tailed Wilcoxon signed-rank tests, chosen due to the limited sample size and non-normal data distribution. Bonferroni correction was used to adjust p-values to account for multiple electrode-wise comparisons, and paired Cohen d was computed to quantify between-condition effect sizes, with thresholds of 0.2, 0.5, and 0.8 corresponding to small, medium, and large effects, respectively.

### 3.1 MRCP

**Figure 2:**
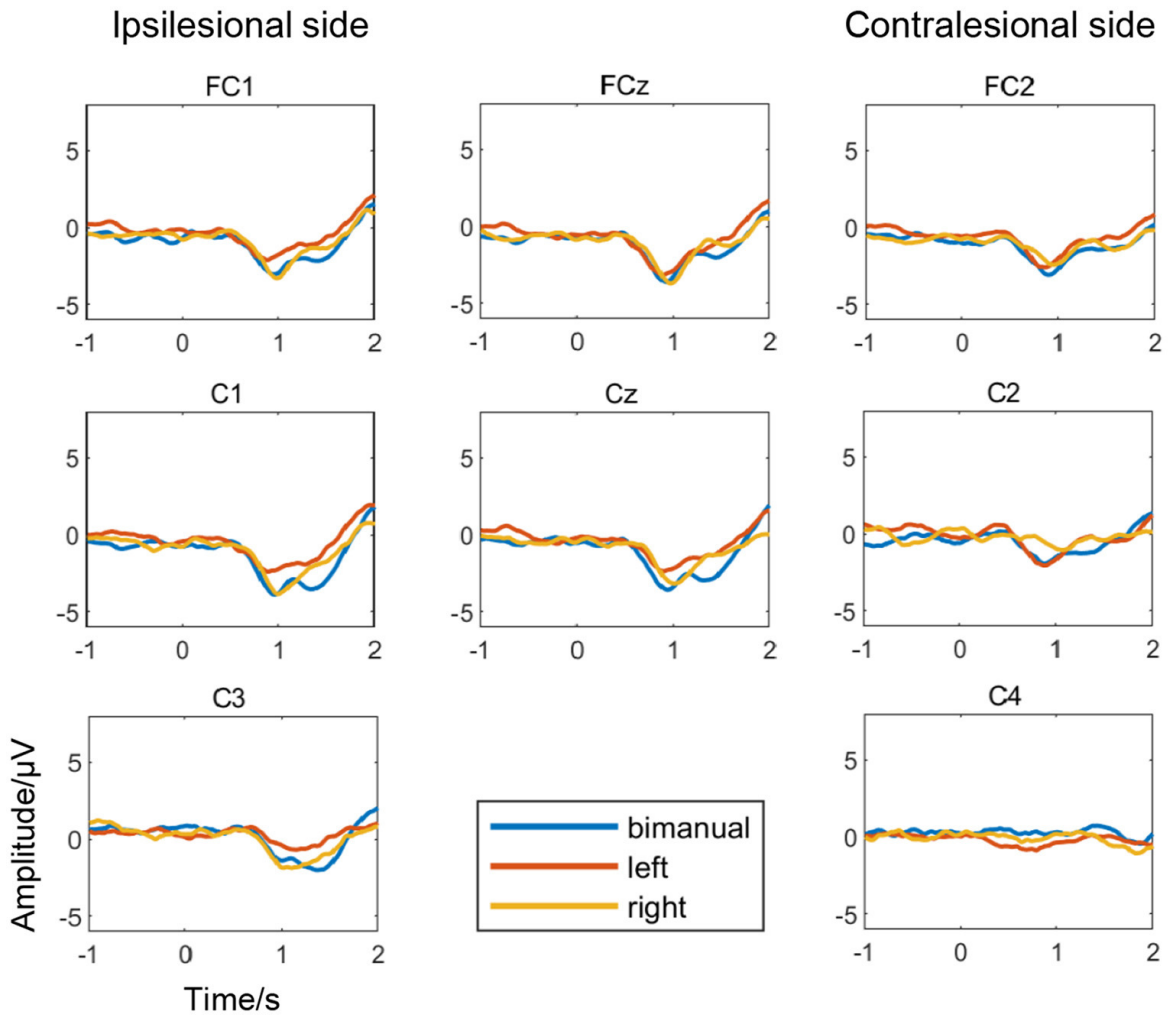
The MRCPs of patient 8 at fronto-central electrodes from –1 s to 2 s under three types of hand movements. The times 0 s refers to movement cue onset, i.e., the 4th second of a trial.

Figure 2 shows the MRCPs of patient 8 with three types of hand movements at the fronto-central region. The time point 0 s refers to the movement cue onset, i.e., the 4 s of a trial. As shown in Figure 2, at around 0.5 s, MRCPs showed a trend of negative offset, then rapidly decreased, and reached the minimum peak negativity (MPN) at around 0.8 s. The blue line represents the MRCPs of bimanual movement, and the red and yellow lines represent the MRCPs of left-hand (unaffected) and right-hand (affected) movements, respectively. Similar MRCPs were mapped for all eight patients (as shown in Supplementary Figures S1–S7).

Table 2 displays the average MPNs of MRCPs elicited by bimanual, unaffected hand, and affected hand movements across all patients. Among the eight channels used for analysis, it was observed that bimanual movement elicited a greater MPN amplitude than either unaffected hand movement or affected hand movement. Given that these eight channels encompass the central midline area and both hemispheres, this phenomenon suggested widespread brain involvement. Wilcoxon signed-rank tests with Bonferroni correction revealed no statistically significant differences for C1 and C2 electrodes (adjusted *p*>0.05), with small-to-medium effect sizes ranging from *d* = 0.26 to *d* = 0.49. These consistent amplitude shifts reflect a uniform neural activation tendency rather than statistically reliable separation between movement types. Although this difference was not statistically significant, it still serves as a promising candidate feature to improve movement decoding accuracy.

**Table 2:** Average minimum peak negativity of MRCP across patients under different hand movements.

Movements	Minimum peak negativity (μV)							
	FCz	FC1	FC2	Cz	C1	C2	C3	C4
Bimanual	–3.068	–2.054	–2.894	–3.016	–1.798	–2.539	–1.489	–1.672
Left (unaffected)	–2.278	–1.679	–2.114	–1.711	–1.102	–1.203	–1.386	–0.844
Light (affected)	–2.257	–1.227	–1.973	–2.438	–1.647	–1.585	–1.253	–1.426

### 3.2 ERD

**Figure 3:**
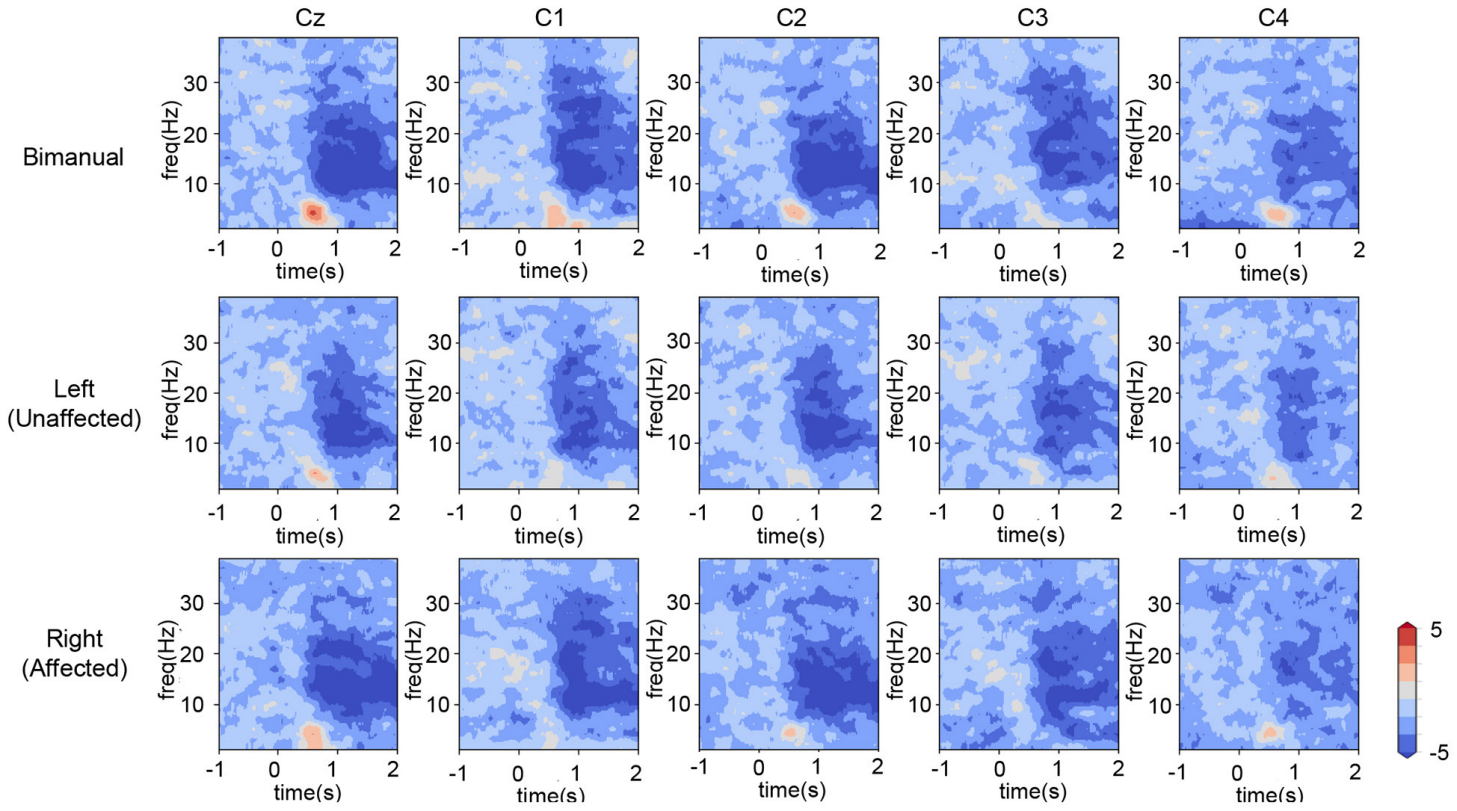
The ERSP of patient 8 from –1 s to 2 s under three types of hand movements at electrodes C1, C2, Cz, C3, and C4. The times 0 s refers to movement cue onset, i.e., the 4th second of a trial.

Figure 3 shows the time-frequency oscillations of three types of hand movements of patient 8. We found a significant ERSP around 0.6 s after the appearance of the movement cue. Furthermore, three types of hand movements showed significant ERSP in both hemispheres, mainly concentrating in the [10–20] Hz frequency band (covering alpha and beta rhythm). Similar figures were mapped for all 8 patients (as shown in Supplementary Figures S8–S14).

The ERDs of different subjects appeared in different frequency bands. From Supplementary material, it was seen that there was a significant difference in ERDs between patients. We found that patients ERDs mainly appeared in the [8–30] Hz frequency band, and the bandwidth of the ERD concentration varied among patients with different lesion locations and severity of brain lesion.

Table 3 displays the average MPNs of ERDs elicited by bimanual, unaffected hand, and affected hand movements across all patients. As shown in Table 3, bimanual movement elicited a greater MPN amplitude than either unaffected hand movement or affected hand movement in C1 and C2, and bimanual movement had greater MPN amplitude than the affected hand movement in Cz. *Post-hoc* Wilcoxon signed-rank tests with Bonferroni correction confirmed significant differences between bimanual and unaffected-hand movement, as well as between bimanual and affected-hand movement at all three electrodes (all adjusted *p* < 0.05). Effect sizes ranged from medium to large (Cohen's *d* = 0.65–0.90), verifying that bimanual coordination evokes markedly stronger oscillatory suppression over bilateral sensorimotor regions. This distinction might contribute to the decoding of bimanual movement.

**Table 3:** The average minimum peak negativity of ERD across patients under different hand movements.

Movements	Minimum peak negativity		
	Cz	C1	C2
bimanual	–0.597	–0.721	–0.624
Left (unaffected)	–0.602	–0.653	–0.600
Right (affected)	–0.529	–0.540	–0.503

### 3.3 Binary classification performance

Table 4 shows the binary classification results (i.e., Rest VS Attempted Movements) by using temporal and spectral features with the LDA classifier from both-hemisphere EEG signals. We calculated the decoding accuracy using a shifted window with a length of 1 s and a step size of 0.1 s from the epoch [–2, 4] s (differing from microstate analysis). We used the center time point of each window to represent this window. The peak time window with the highest decoding accuracy was selected independently for each patient. The average decoding accuracies of decoding models based on temporal and spectral features for three types of hand movement peaked at 1.18, 0.94, 1.06, and 1.26, 1.70, 1.76s, respectively, and were 87.51%± 3.39%, 79.57%± 7.31%, 83.84%± 6.80%, and 82.85%± 6.63%, 72.85%± 8.26%, 70.98%± 6.54%, respectively. We found that the average peak accuracy of bimanual movement was about 4% higher than that of the affected-hand movement when using temporal features, although Wilcoxon test didn't show a significant difference (*p*=0.3125). Besides, the average peak accuracy of bimanual movement was higher than that of the unaffected-hand movement by 8%, and Wilcoxon test showed that the difference was significant (*p* = 0.0160). For the spectral features, the average peak accuracy of bimanual movement was about 12% higher than that of the affected-hand movement, and Wilcoxon test showed that the difference was significant (*p* = 0.0160). The average peak accuracy of bimanual movement was higher than that of the unaffected-hand movement by 10%, Wilcoxon test showed that the difference was significant (*p*=0.0080). We performed a trial-level permutation test across 1000 iterations. The mean chance-level accuracy was 50.12% ± 1.86%, closely matching the theoretical random level. To address multiple tests involving different movement conditions, feature types, time windows, channels and frequency bands, all p-values were adjusted via Bonferroni correction. After correction, the comparisons between bimanual and unaffected-hand movements yielded *p* = 0.016 for temporal features and *p* = 0.008 for spectral features, while the comparisons between bimanual and affected-hand movements returned *p* = 0.008 and *p* = 0.016 correspondingly. The paired Cohen d was 0.81 with 95% CI [0.36, 1.25] for bimanual vs. unaffected-hand movements, and 0.63 with 95% CI [0.22, 1.04] for bimanual vs. affected-hand movements. In the spectral feature analysis, the corrected p-value was 0.008 when comparing bimanual movements with unaffected-hand movements and 0.016 when compared with affected-hand movements, with a paired effect size of *d* = 0.92 [95% CI (0.47, 1.37)] and *d* = 1.01 [95% CI (0.55, 1.46)], respectively. These results confirm that bimanual motor attempts achieve evidently higher decoding performance than unimanual motor attempts.

**Table 4:** Average peak performance comparisons of all patients using temporal and spectral features.

Patient No.	Temporal						Spectral					
	Peak accuracy (%)			Peak time (s)			Peak accuracy (%)			Peak time (s)		
	Bimanual	Unaff.	Aff.	Bimanual	Unaff.	Aff.	Bimanual	Unaff.	Aff.	Bimanual	Unaff.	Aff.
P1	84.96	81.06	86.00	1.9	0.8	0.6	76.32	65.15	64.25	0.7	1.1	1.2
P2	89.01	83.31	81.84	2.6	1.8	3.6	89.06	82.81	74.18	2.7	3.9	3.7
P3	86.26	73.69	86.54	0.8	0.6	1.0	80.59	64.73	72.91	1.0	0.9	1.0
P4	88.24	90.04	88.30	0.8	0.8	0.5	77.36	76.02	81.81	0.8	1.9	1.1
P5	90.69	72.44	79.45	0.9	1.0	0.4	93.70	66.33	68.03	2.5	2.7	3.4
P6	90.56	86.96	94.51	0.9	0.8	0.9	86.29	79.85	75.01	0.8	0.9	1.9
P7	80.73	69.15	71.47	0.7	0.8	0.7	84.32	82.60	61.11	0.7	1.1	0.9
P8	89.59	79.87	82.63	0.8	0.9	0.8	75.12	65.30	70.52	0.9	1.1	0.9
Mean ± Std	87.51 ± 3.39	79.57 ± 7.31	83.84 ± 6.80	1.18 ± 0.69	0.94 ± 0.37	1.06 ± 1.04	82.85 ± 6.63	72.85 ± 8.26	70.98 ± 6.54	1.26 ± 0.83	1.70 ± 1.08	1.76 ± 1.15

Unaff., unaffected; Aff., affected.

**Figure 4:**
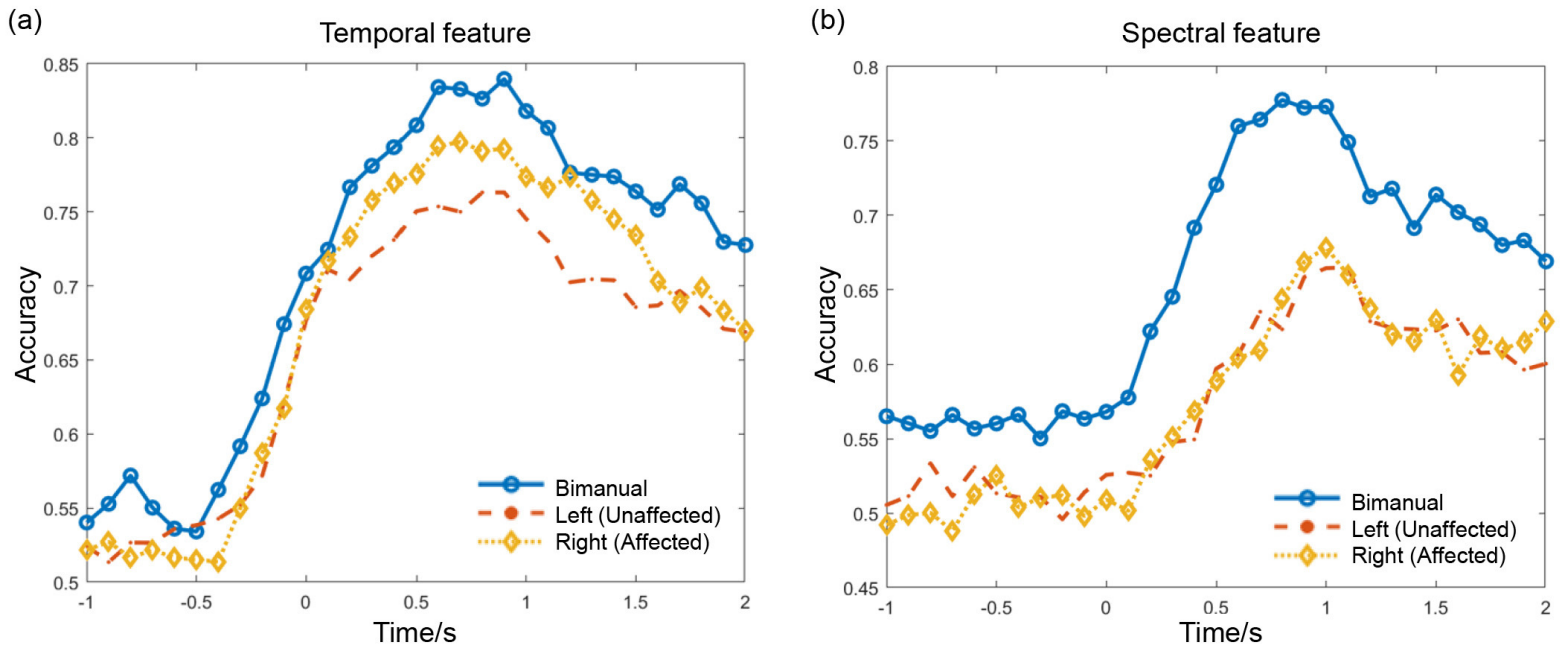
The grand average decoding performance across all patients under three types of hand movements using **(a)** temporal features and **(b)** spectral features. The continuous decoding accuracy was obtained on shifted windows with the window length of 1 s and step size of 0.1 s from –1.0 s to 2.0 s of movement cue onset.

Figure 4 shows the average continuous decoding accuracy for three types of hand movements by using different features across all patients. During the period from –1.0 s to 2.0 s of the movement cue onset, there were 31 windows. We used the center time point of each window to represent this window. In the window of [0, 1] s, average accuracies of three types of hand movements were above the chance level, and the time point of actual motor attempt onset was about 0.8 s (according to the results of MRCPs), indicating that the movement intention can be decoded before actual movement onset. Besides, the average decoding accuracy of bimanual movement was higher than that of the affected-hand movement after movement cue onset.

These results indicated that the BCI decoding performance of bimanual movements was not only higher than that of affected-hand movements, but also higher than that of unaffected-hand movements. This could be attributed to that when involving both the affected hand and unaffected hand in bimanual movements, there were more brain activations compared to unilateral hand movements. Consequently, these results implied that bimanual movement might be a promising candidate paradigm for the rehabilitation of stroke patients based on BCIs.

## 4 Discussion and conclusion

In this paper, to explore whether bimanual movement contributes to the movement intentions' decoding of stroke patients based on EEG signals, we studied the neural signatures and decoding under three types of hand movements (i.e., bimanual movement, unaffected-hand unimanual movement, affected-hand unimanual movement) using EEG signals in time and frequency domains. Neural signatures were represented in forms of the MRCP and ERD. The decoding models of movement intention were built and tested under three movements for comparisons. This work may be beneficial in promoting the rehabilitation of patients with impaired hand function.

Although there were differences in MRCPs among patients with different lesion locations and severities, three patients showed greater MRCPs for bimanual movements than for unimanual movements in both hemispheres. Meanwhile, five out of eight patients showed greater MRCPs for bimanual movements than for unimanual movements in the midline area of the cortex. These results indicated that neural activations of each hand could contribute to the decoding of bimanual movements. Bimanual motor attempts fully recruit the bilateral motor networks of both cerebral hemispheres, rather than the unilateral cortical circuits activated during single-hand movements. Meanwhile, synchronized bimanual motor planning reinforces interhemispheric interaction via transcallosal pathways, which further amplifies movement-related neural activities across sensorimotor regions.

ERD was subject-specific, and the ERD of different subjects appeared in different frequency bands (Pfurtscheller and da Silva, 1999). Furthermore, due to differences in the lesion locations and severities, the differences in ERD between patients may be even more pronounced. We found that the ERD of patients mainly appeared in the [8–30] Hz frequency band (including alpha and beta rhythms), and the ERD of bimanual movement was greater than that of the unimanual movement in specific frequency bands and electrodes for each patient. The bandwidth of the ERSP concentration also varied among patients with different locations and severities of brain lesion. The ERSP of Patients 1 and 2 was similar and mainly concentrated in the [10–15] Hz frequency band. The ERSP of Patients 3 and 6 mainly concentrated in the [15–20] Hz frequency band. The ERSP of Patients 5 and 8 mainly concentrated in the [10–20] Hz frequency band. The ERSP of Patients 4 and 7 mainly concentrated in the [8–20] Hz and [8–10] Hz frequency bands, respectively. Despite such inter-individual differences in dominant frequency bands, the consistently enhanced ERD during bimanual movements further proves that bilateral tasks induce more intensive cortical oscillation modulation across the bilateral motor network.

The neural signature results suggested that brain activation was more pronounced for bimanual movements. The lesion-ipsilateral hemisphere in bimanual movement was more active than that in the unimanual movement. For hemiplegic stroke patients, the unaffected hemisphere generally generates compensatory activation to compensate for motor dysfunction of the lesioned side (Staines et al., 2001; Stewart et al., 2006). During bimanual tasks, the active participation of the unaffected hand further strengthens such compensatory cortical activation. More importantly, the activated unaffected hemisphere can deliver excitatory signals to the damaged cortex through interhemispheric pathways, producing an obvious neural facilitation effect on the lesion-ipsilateral hemisphere. The findings were consistent with a basic assumption of the use of bilateral movement therapy in Staines et al. (2001); Stewart et al. (2006); Carson (2005), showing that bilateral movements might increase the activation of the lesion-ipsilateral hemisphere caused by the involvement of the lesion-contralateral hemisphere.

The experimental results showed that the decoding performance of bimanual movements was significantly higher than that of the unimanual movement. The improved decoding performance of bimanual movements is driven by the combined effects of enhanced MRCP components, distinct ERD patterns and broader bilateral cortical activation, rather than a single neural characteristic. On the one hand, the amplified low-frequency MRCP signals during bimanual movements provide more prominent time-domain features. The larger negative amplitudes of MRCP clearly distinguish motor attempts from the resting state, laying a solid foundation for high-accuracy classification based on temporal features. On the other hand, bimanual tasks generate stronger ERD responses within the 8–30 Hz frequency band. Even though the dominant frequency of ERD varies across individual patients, these enhanced oscillatory patterns form more discriminative spectral features. Benefiting from the FBCSP algorithm, subject-optimized frequency band features can be fully extracted, which further improves classification performance. In addition, bimanual movement activates a wider range of bilateral sensorimotor cortices. The expanded spatial distribution of neural activities enriches valid EEG information for decoding models, which further stabilizes classification results. Meanwhile, the increased cognitive and physical effort demanded by simultaneous bilateral motion may also enlarge the separability of EEG signals between movement classes. These plausible underlying mechanisms are all presented and balanced in our interpretation of the bimanual decoding benefit.

The decoding models constructed using time-domain and frequency-domain features achieved average peak decoding accuracy of 87.51% and 82.85%, respectively. The negative offset amplitudes of MRCP can serve as effective features for decoding movement intention. Furthermore, ERD has subject specificity, and the ERD of different subjects appears in different frequency bands (Shakeel et al., 2015). By using FBCSP, the optimal frequency band features for each subject can be extracted. As shown in Figure 4, the average decoding accuracy of bimanual movements was significantly higher than that of unimanual movements in terms of temporal and spectral features. By comparing the average decoding accuracy between bimanual and unimanual movements, better accuracy was obtained in bimanual movements, with 4% to 12% higher than in unimanual movements. Besides, the decoding accuracy higher than the chance level (50%) was observed at 0.5 s (i.e., the window of [0 1] s). The actual motor attempt time onset was about 0.8 s, and so the movement intention was included in this window, indicating that the movement intention can be decoded. With the shifted windows, the EEG signals contained more useful information related to hand motor attempts, improving decoding accuracy. The decoding accuracy of bimanual movements was higher than that of affected-hand movements and unaffected-hand movements, which may indicate that better motor control facilitated by BCIs could potentially aid in the rehabilitation of stroke patients. However, we would like to clarify that the study does not establish a direct causal relationship between decoding accuracy and rehabilitation effects. Future work should focus on correlating decoding performance with clinical rehabilitation measures to further understand the potential therapeutic benefits of BCIs. The bimanual paradigm can be readily integrated into practical BCI rehabilitation systems. It yields more stable EEG control signals to operate robotic gloves and functional electrical stimulation devices. Notably, this approach works well for patients with weak motor intention signals in the affected hand, as bilateral activation compensates for insufficient neural responses in the lesioned hemisphere and improves decoding reliability. This integrated solution can support routine rehabilitation training and promote hand function recovery. In addition, it was found that patients tended to save more physical effort when executing bimanual movements through an oral questionnaire survey. This work provides a potential candidate paradigm for patients hand function neurorehabilitation.

In summary, this work investigated EEG neural signatures and movement decoding of bimanual and unimanual motor attempts in stroke patients with hemiplegia. Bimanual attempts produce more prominent MRCP and ERD activities and evidently improve decoding accuracy, owing to comprehensive bilateral cortical recruitment. The proposed paradigm delivers more stable control signals for BCI rehabilitation devices and benefits patients with poor affected-hand motor intentions. As limitations, the current study has a limited number of patients and obvious inter-individual differences across patients. Another limitation lies in the lack of synchronous kinematic recordings and standalone EMG signals for direct verification of residual movements and muscle activation. In future research, we will integrate multi-modal recordings including kinematic data and independent EMG signals to conduct more comprehensive control analyses and eliminate movement confounding. Future research will expand the sample size, refine decoding methods, integrate multi-modal recordings including kinematic data and independent EMG signals to conduct more comprehensive control analyses and eliminate movement confounding, and carry out clinical trials to further verify the rehabilitation efficacy of this approach.

## Data Availability

The raw data supporting the conclusions of this article will be made available by the authors, without undue reservation.
