# Supplementary material for: Bimanual attempts improving EEG-based hand movement decoding in stroke patients with hemiplegia
Source: Front Hum Neurosci. 2026 Jul 28;20:1890774. doi: 10.3389/fnhum.2026.1890774 (PMC13457473; doi:10.3389/fnhum.2026.1890774)
Supplement: Supplementary file 1 [file Data_Sheet_1.pdf]

Supplementary Material

1 MRCPS

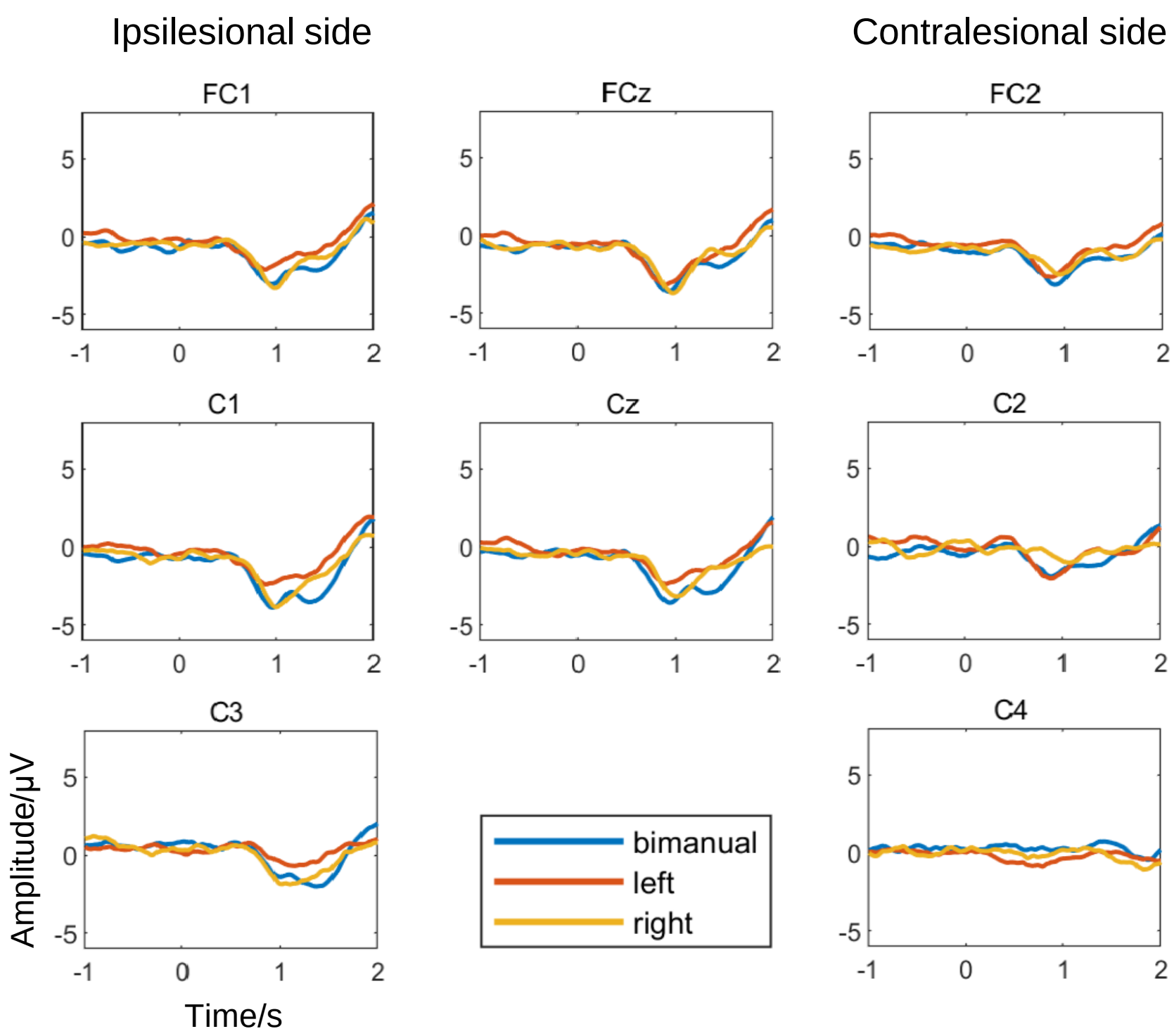

Figure S1. The MRCP of patient 1 at fronto-central electrodes from -1 s to 2 s under three types of hand movements.

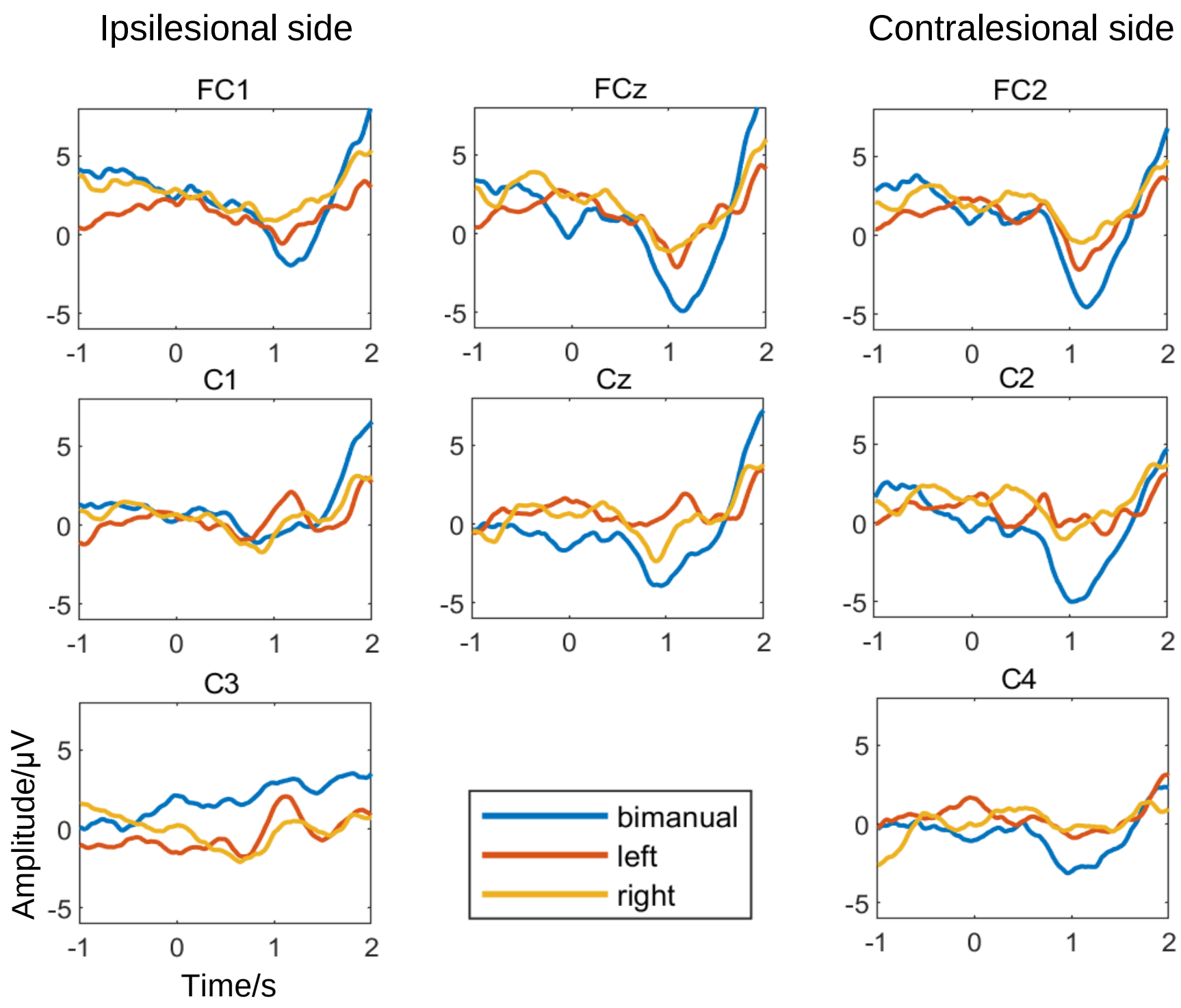

Figure S2. The MRCP of patient 2 at fronto-central electrodes from -1 s to 2 s under three types of hand movements.

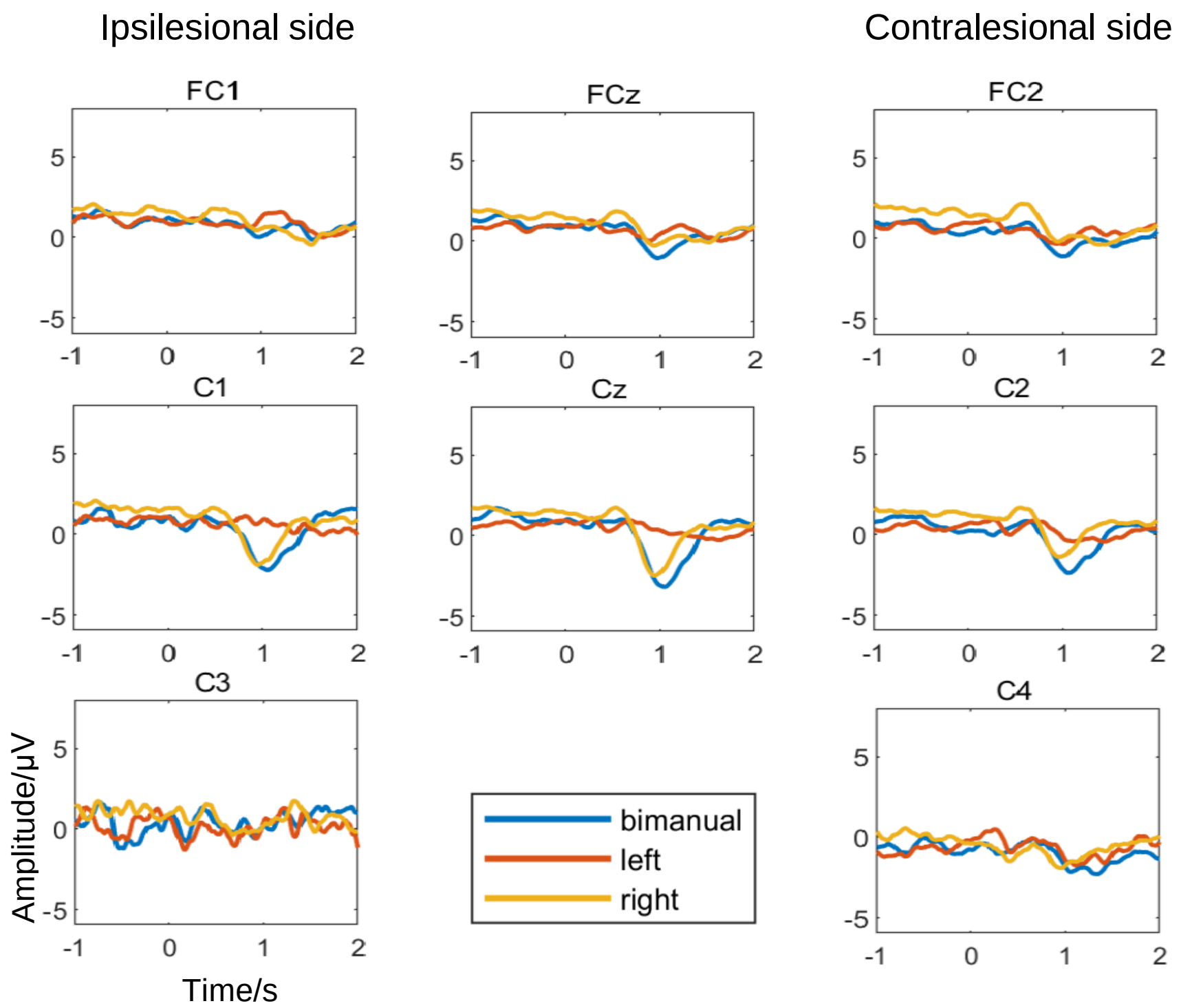

Figure S3. The MRCP of patient 3 at fronto-central electrodes from -1 s to 2 s under three types of hand movements.

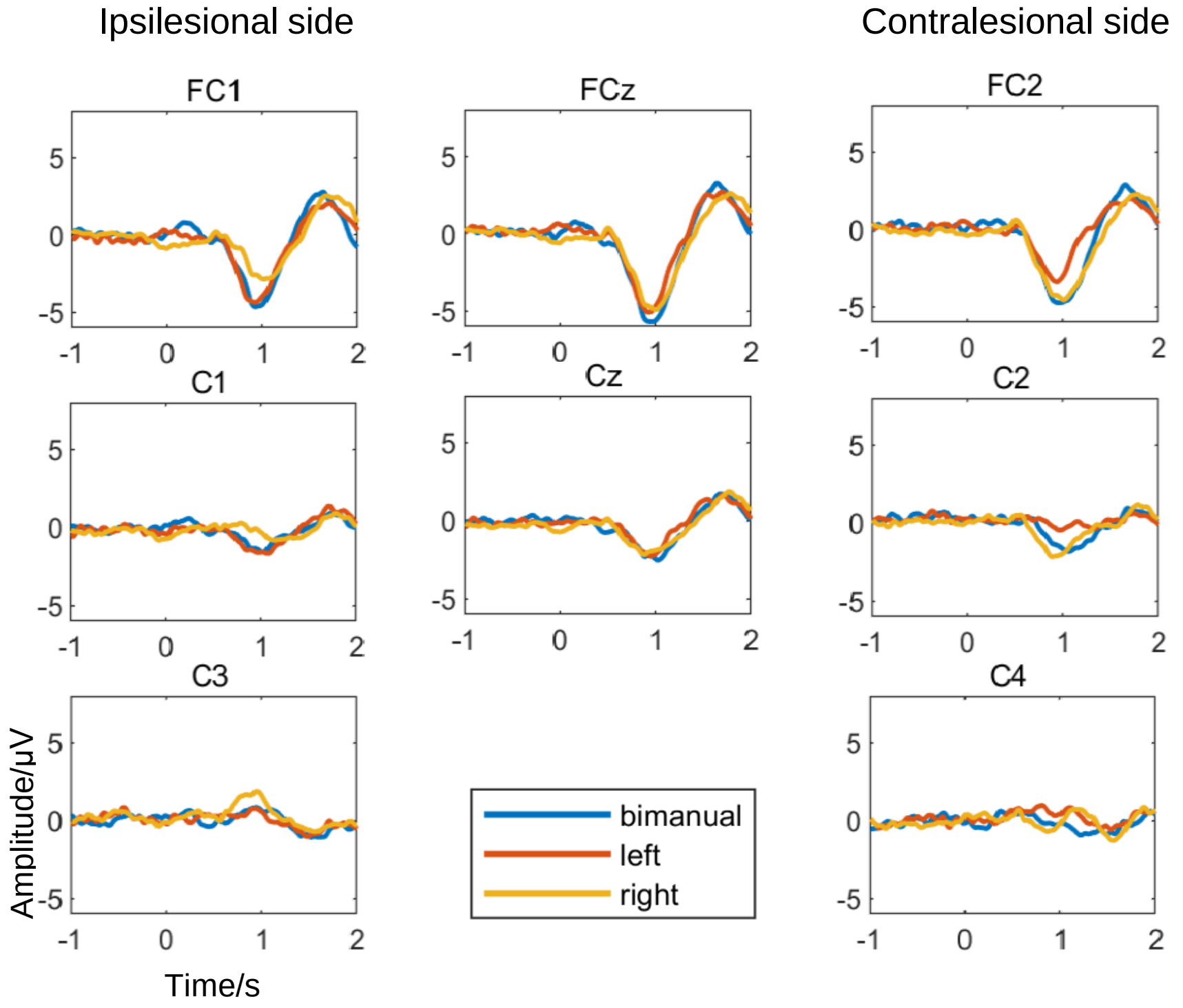

Figure S4. The MRCP of patient 4 at fronto-central electrodes from -1 s to 2 s under three types of hand movements.

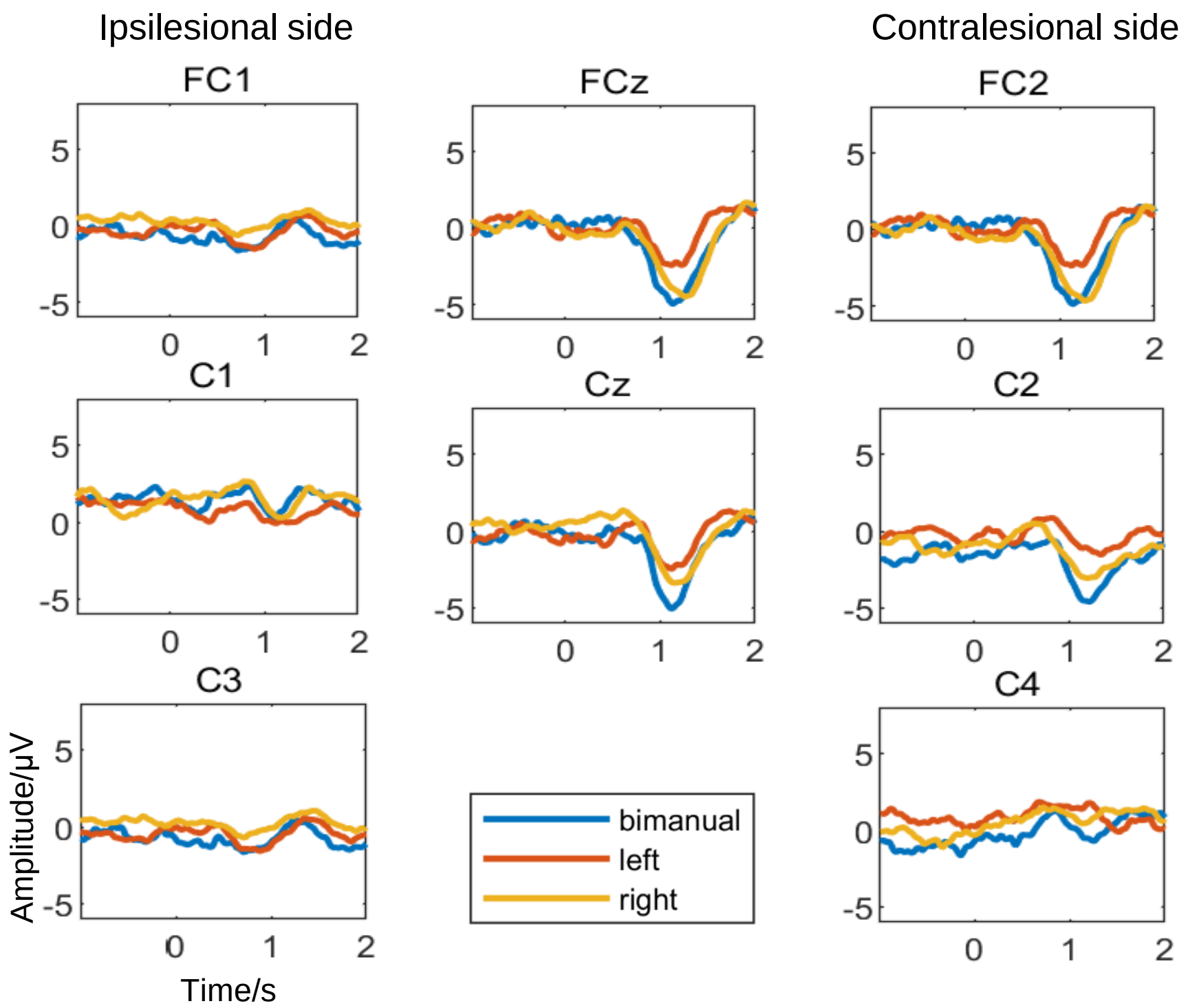

Figure S5. The MRCP of patient 5 at fronto-central electrodes from -1 s to 2 s under three types of hand movements.

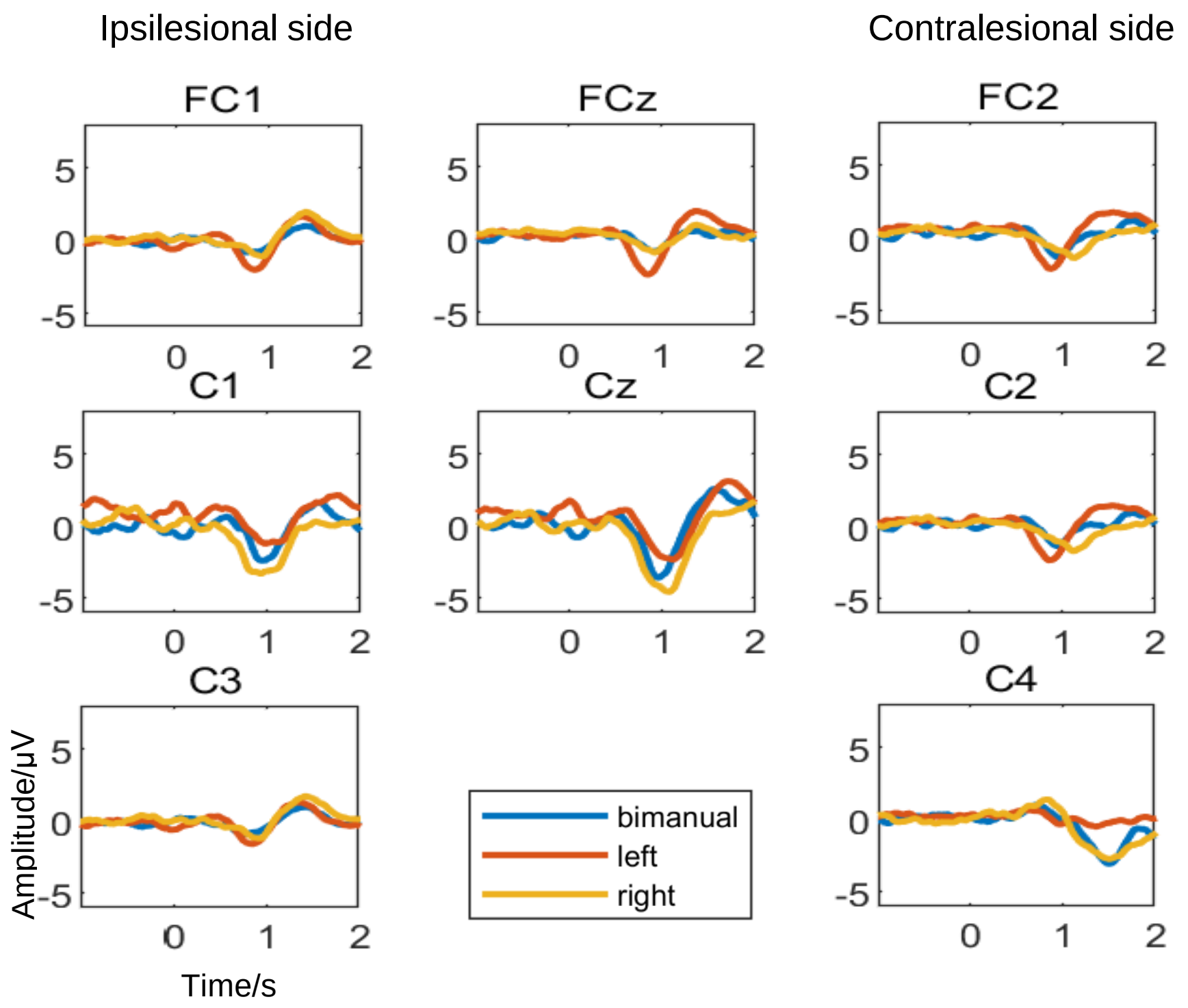

Figure S6. The MRCP of patient 6 at fronto-central electrodes from -1 s to 2 s under three types of hand movements.

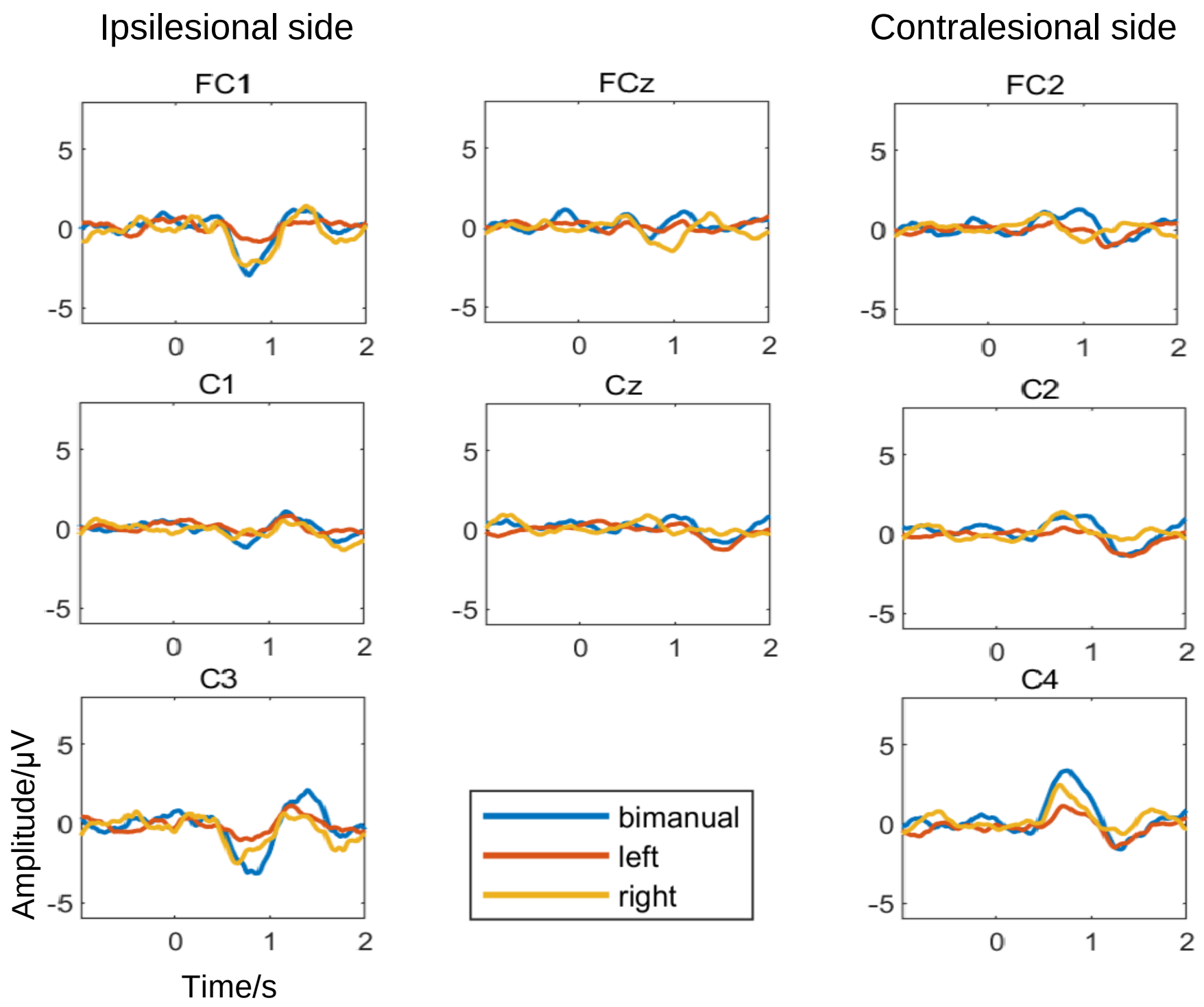

Figure S7. The MRCP of patient 7 at fronto-central electrodes from -1 s to 2 s under three types of hand movements.

2 ERDS

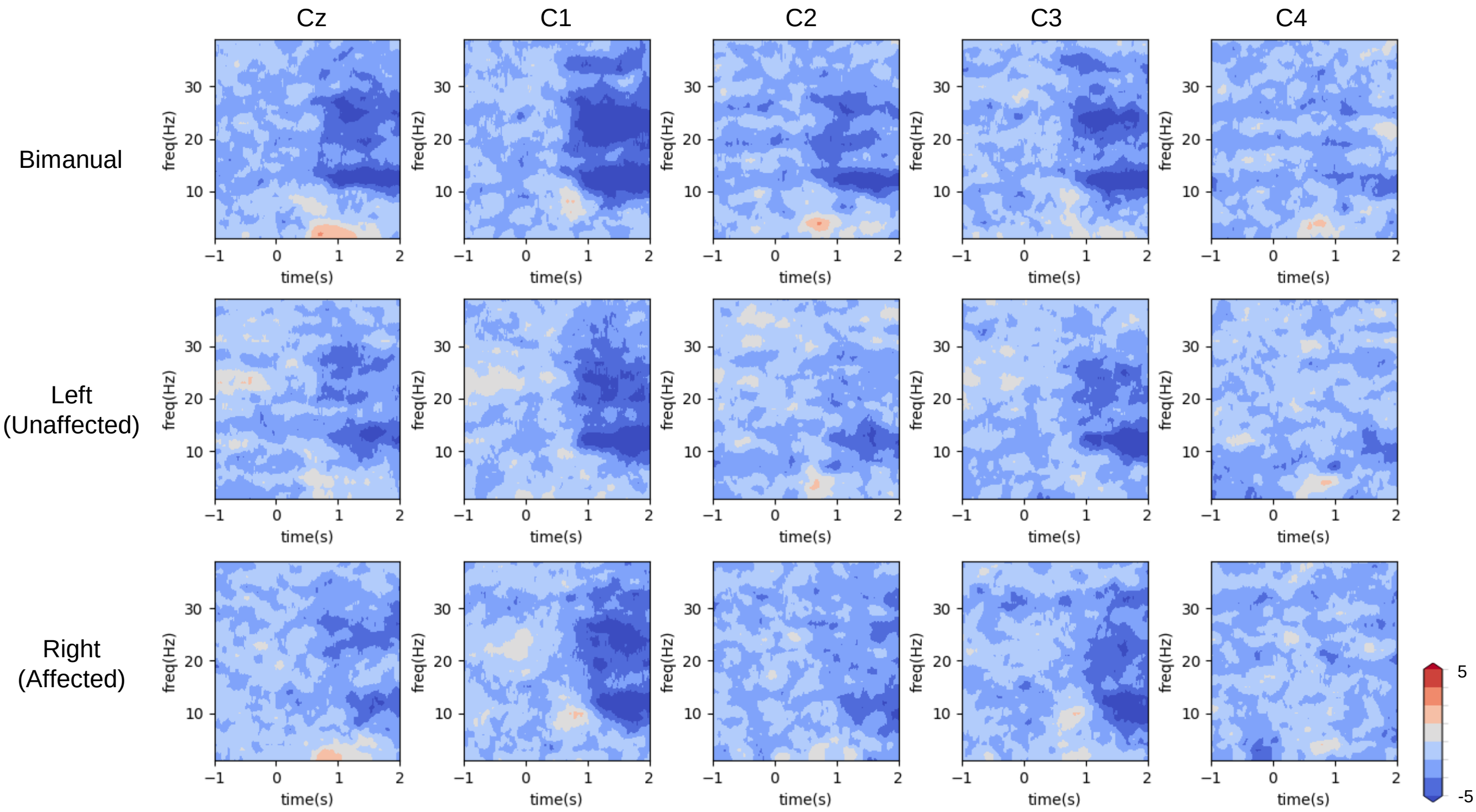

Figure S8. The ERSP of patient 1 from -1 s to 2 s under three types of hand movements at electrodes C1, C2, Cz, C3, and C4.

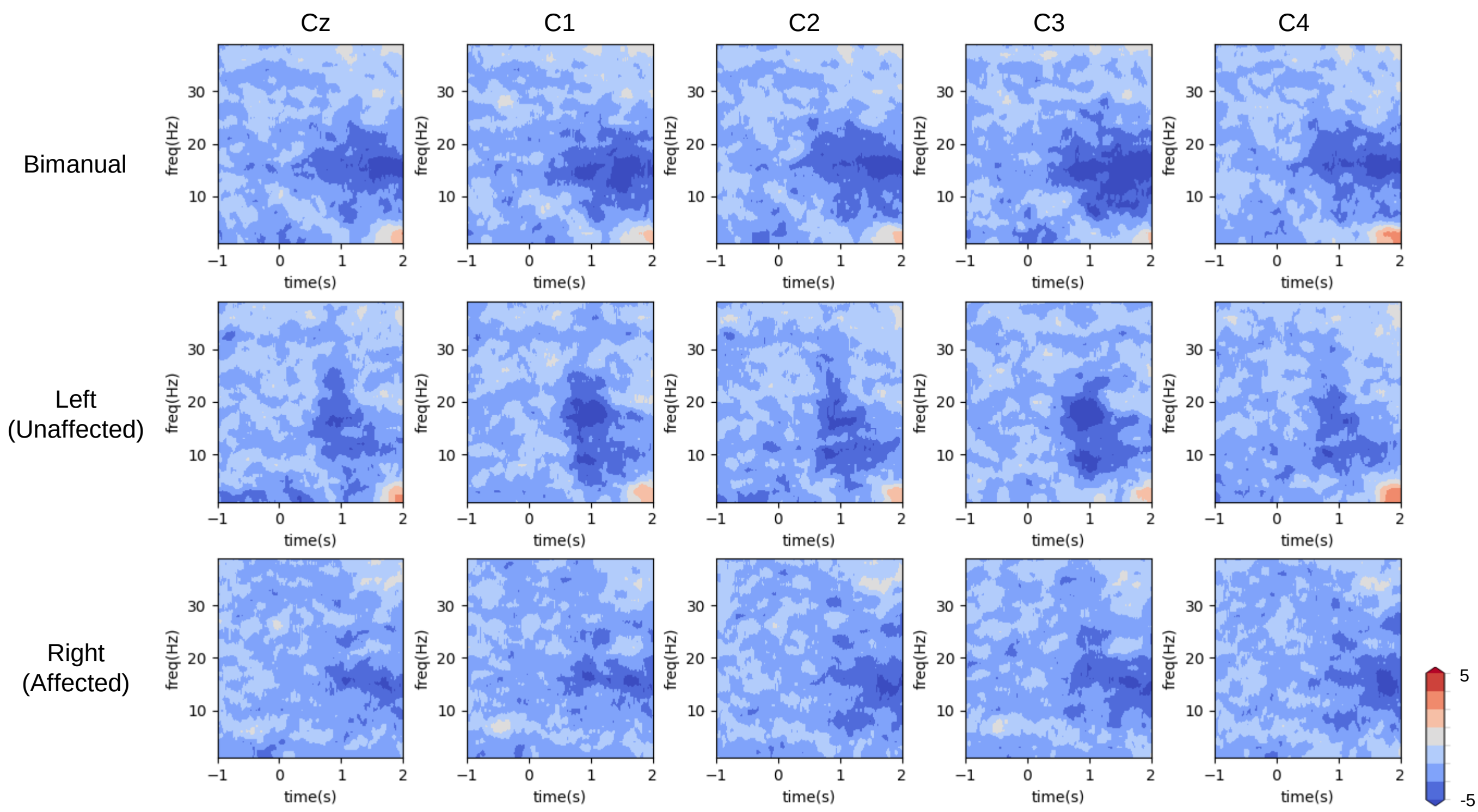

Figure S9. The ERSP of patient 2 from -1 s to 2 s under three types of hand movements at electrodes C1, C2, Cz, C3, and C4.

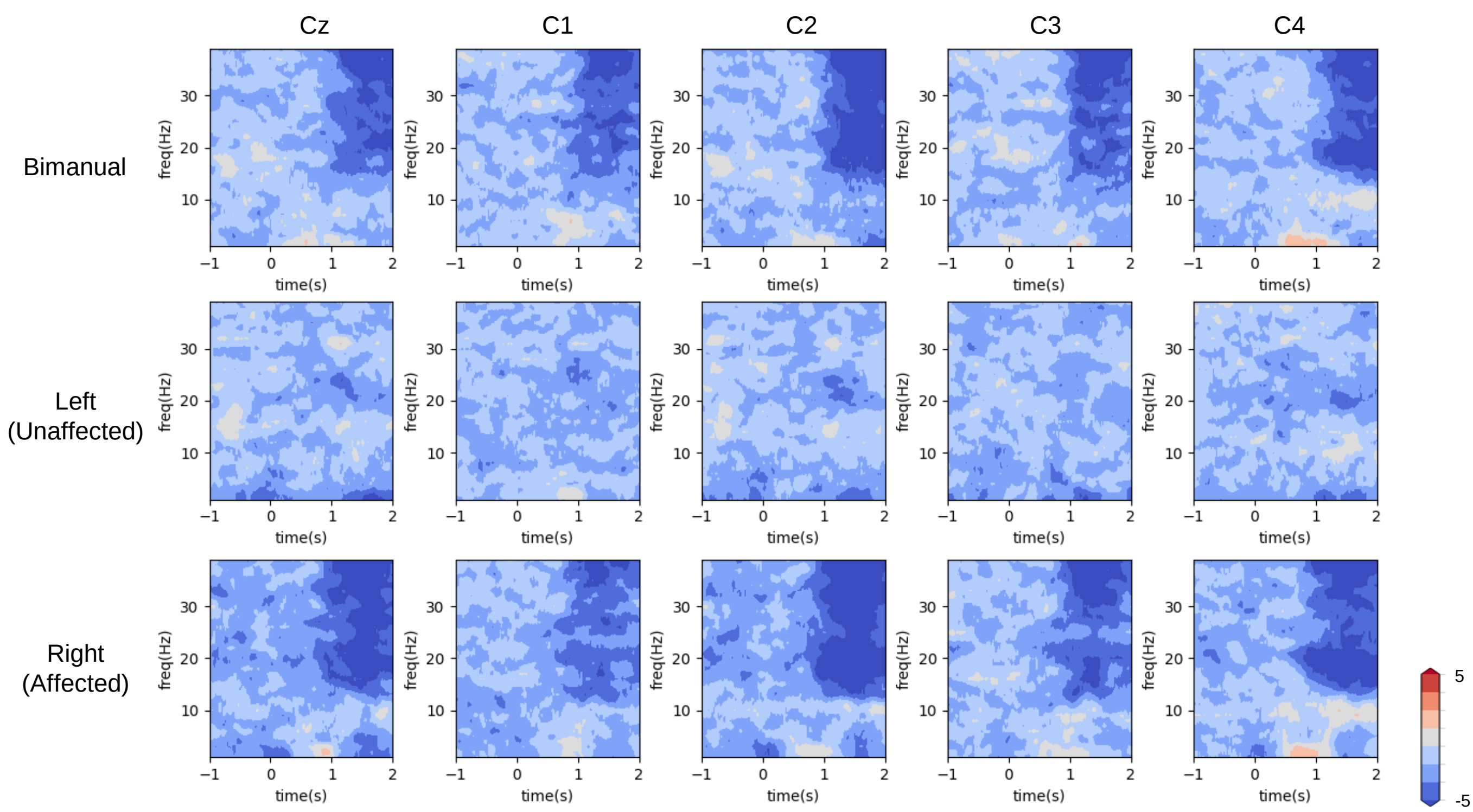

Figure S10. The ERSP of patient 3 from -1 s to 2 s under three types of hand movements at electrodes C1, C2, Cz, C3, and C4.

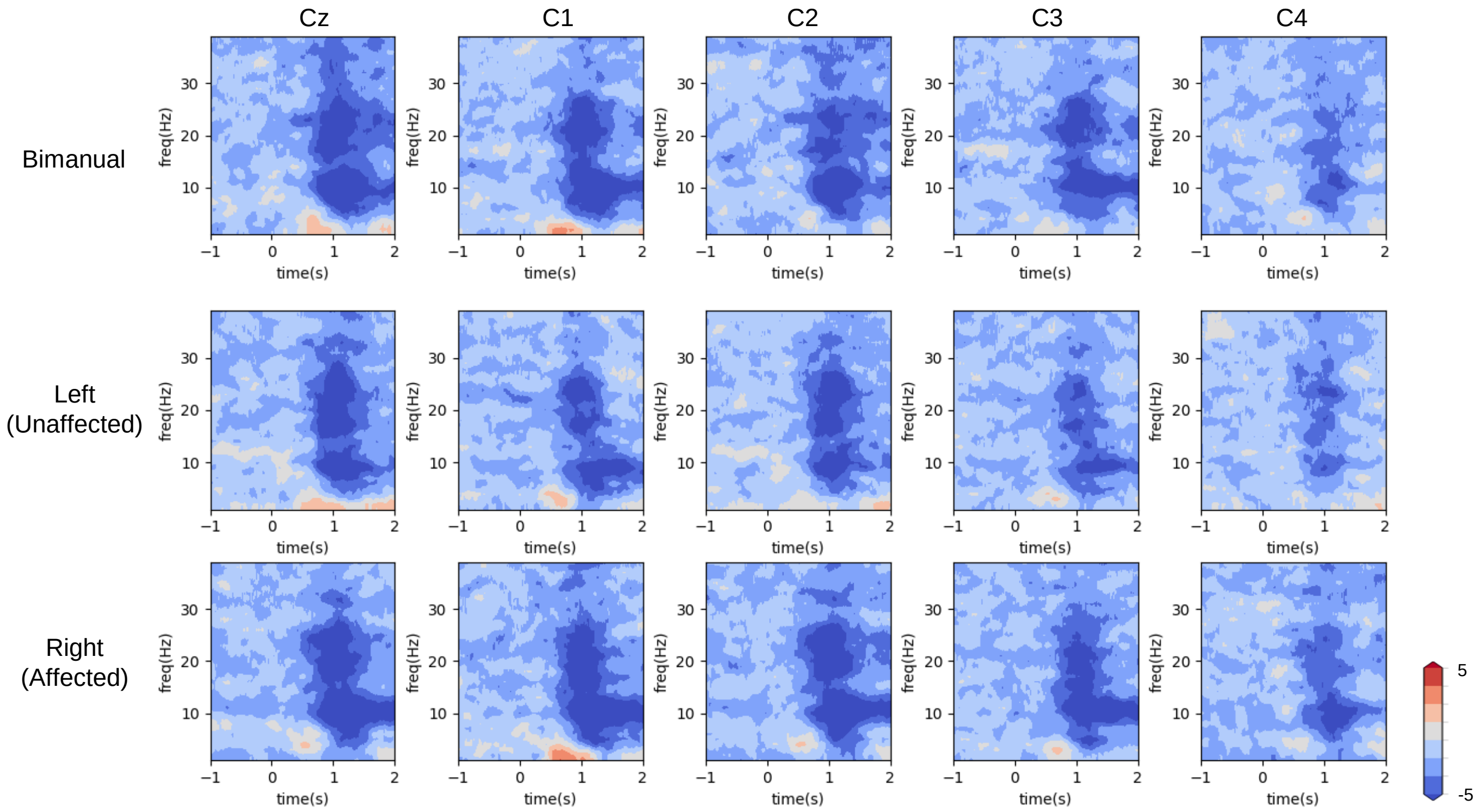

Figure S11. The ERSP of patient 4 from -1 s to 2 s under three types of hand movements at electrodes C1, C2, Cz, C3, and C4.

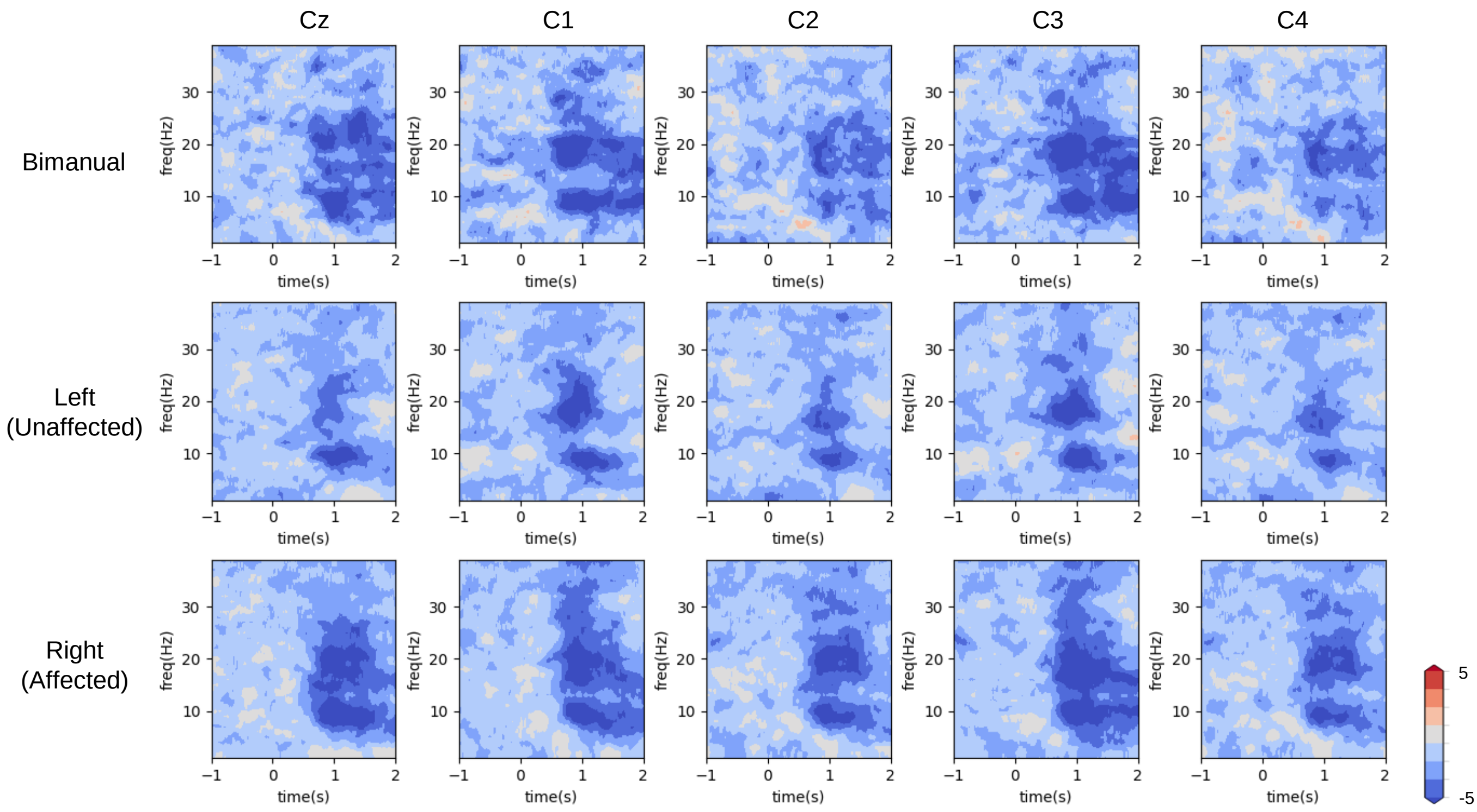

Figure S12. The ERSP of patient 5 from -1 s to 2 s under three types of hand movements at electrodes C1, C2, Cz, C3, and C4.

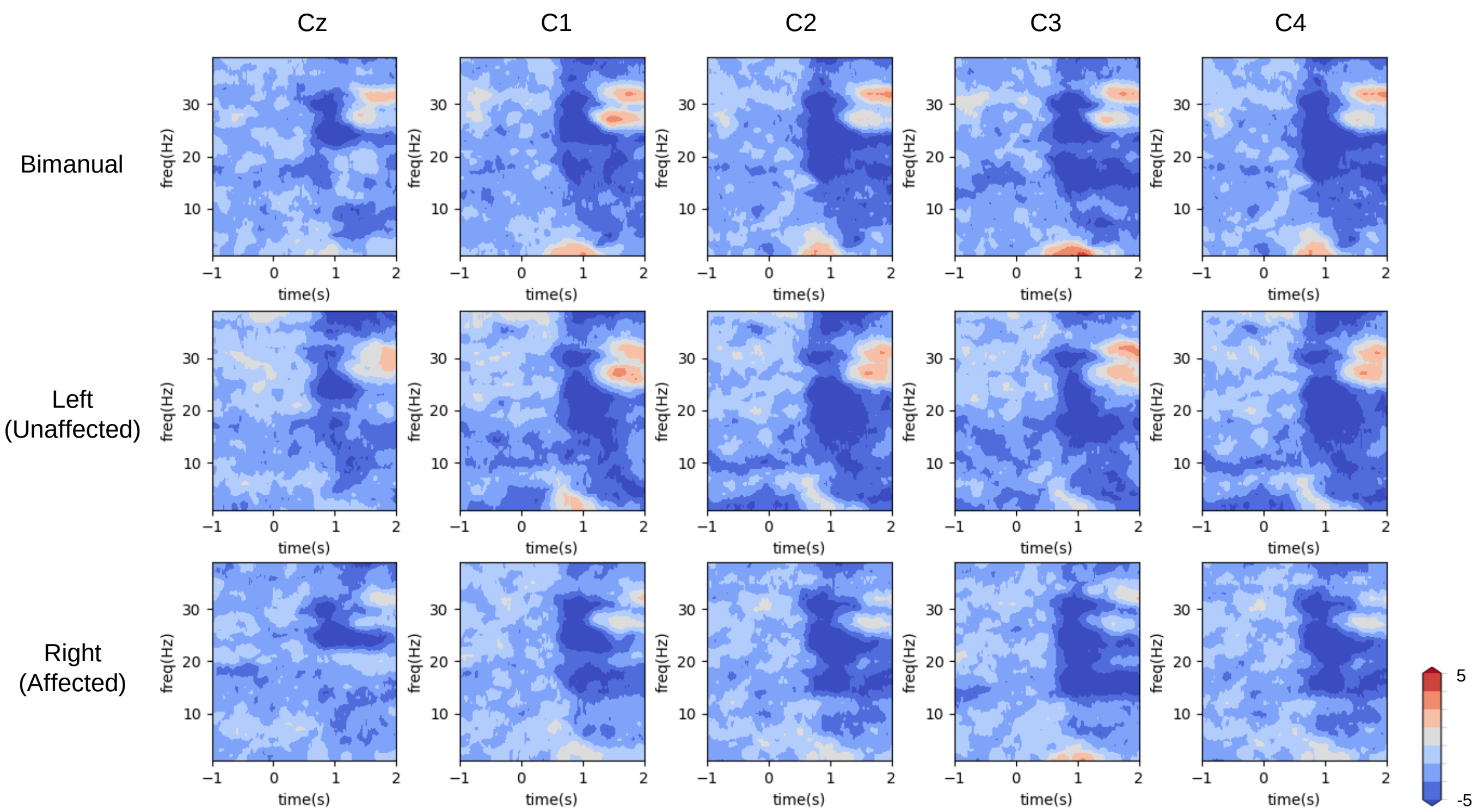

Figure S13. The ERSP of patient 6 from -1 s to 2 s under three types of hand movements at electrodes C1, C2, Cz, C3, and C4.

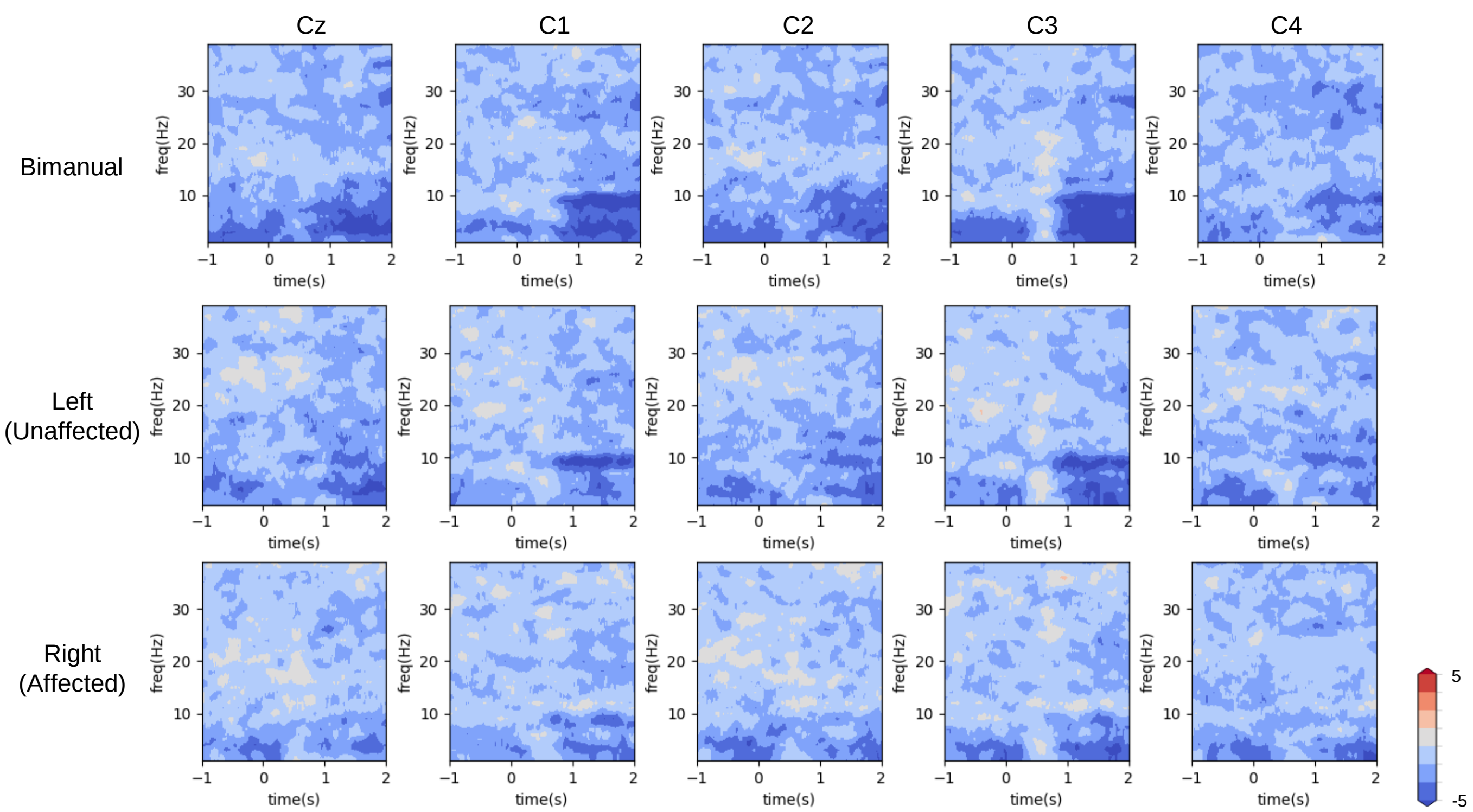

Figure S14. The ERSP of patient 7 from -1 s to 2 s under three types of hand movements at electrodes C1, C2, Cz, C3, and C4.
